# Supplementary material for: Identification of photoperiod-induced specific miRNAs in the adrenal glands of Sunite sheep (Ovis aries)
Source: Front Vet Sci. 2022 Jul 22;9:888207. doi: 10.3389/fvets.2022.888207 (PMC9354845; doi:10.3389/fvets.2022.888207)
Supplement: Supplementary file 1 [file Data_Sheet_1.ZIP › Supplementary file/miRDeep2 file.zip!/miRDeep2 file/Novel_Structure/LP42A2/Novel_87.pdf]

Provisional ID	:	NC_019471.2_24739
Score total	:	2.4
Score for star read(s)	:	-1.3
Score for read counts	:	0
Score for mfe	:	2.1
Score for randfold	:	1.6
Score for cons. seed	:	
Total read count	:	65
Mature read count	:	35
Loop read count	:	0
Star read count	:	30

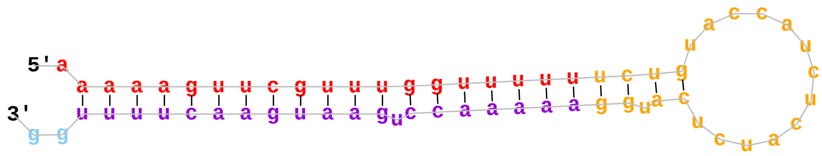

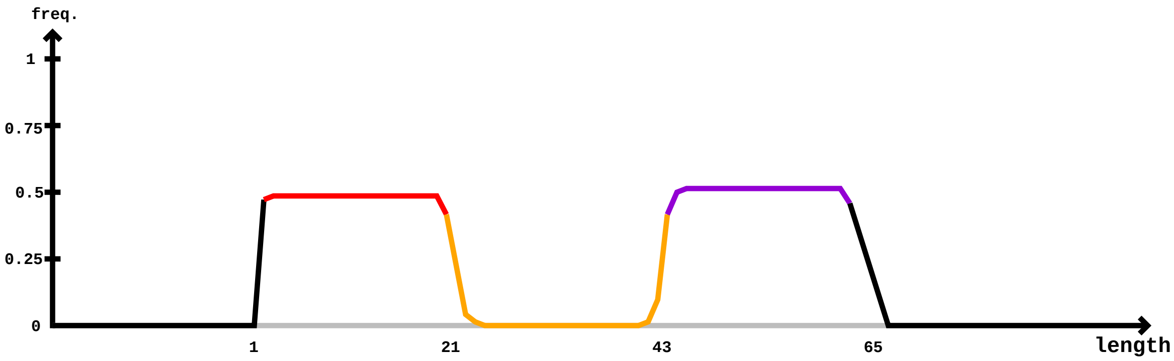

Mature **Star**

```
5'- acuucuguauuggguuggcuaaaaaguucguuugguuuuuucuguaccaucucaucucauggaaaaaccugaaugaacuuuuggccaacccagcagaaauuaucaaggagga  -3'   obs
    acuucuguauuggguuggcuaaaaaguucguuugguuuuuucuguaccaucucaucucauggaaaaaccugaaugaacuuuuggccaacccagcagaaauuaucaaggagga         exp
    ..(((((..(((((((((((((.(((((((((((((((((((((.............)).))))))))).))))))))))))))))))))))))))))..............   reads   mm   sample
    ....................aaaaaguucguuuggGuuu.........................................................................   5       1    seq
    ....................aaaaaguucguuuggGuuuu........................................................................   24      1    seq
    ....................aaaaaguucguuugguuuuC........................................................................   1       1    seq
    ....................aaaaaguucguuugguuuuuu.......................................................................   2       0    seq
    ....................aaaaaguucguuugguuuuuuc......................................................................   2       0    seq
    .....................aaaaguucguuugguuuuuucu.....................................................................   1       0    seq
    ............................................................ggaaaaaccCgaaugaacuuu...............................   1       1    seq
    .............................................................gaaaaaccugaaCgaacuuu...............................   1       1    seq
    .............................................................gaaaaaccugaaugaacuuuA..............................   1       1    seq
    .............................................................gGaaaaccugaaugaacuuuu..............................   1       1    seq
    .............................................................gaaaaaccugaaugaacuuuu..............................   2       0    seq
    .............................................................gaaaaaccCgaaugaacuuuug.............................   1       1    seq
    ..............................................................aaaaaccugaaGgaacuuu...............................   1       1    seq
    ..............................................................aaaaaccugaaugaacuuuu..............................   5       0    seq
    ..............................................................aaaaaccCgaaugaacuuuu..............................   2       1    seq
    ..............................................................aaaaaccugaaCgaacuuuu..............................   5       1    seq
    ..............................................................aaaaaccugaaGgaacuuuu..............................   7       1    seq
    ..............................................................aaaaaUcugaaugaacuuuug.............................   2       1    seq
    ..............................................................aaaaaccugaaugaacuuuuUgc...........................   1       1    seq
    ...............................................................aaaaccugaaugaacuuu...............................   1       0    seq
    ...............................................................aaaaccugaaugaacuuuu..............................   1       0    seq
    ...............................................................aaaaUcugaaugaacuuuug.............................   2       1    seq
    ...............................................................aaaaUcugaaugaacuuuugg............................   1       1    seq
    ...............................................................aaaaccugaaugaacuuuuggG...........................   1       1    seq
    ................................................................aaaccugaaugaacuuuugg............................   1       0    seq
```
